# Periodontal effect of augmented corticotomy-assisted orthodontics versus conventional orthodontics in treatment of adult patients with bialveolar protrusion

**DOI:** 10.1186/s12903-022-02107-3

**Published:** 2022-03-19

**Authors:** Bing Wang, WenQiong Xi, Hui Chen, Jinlong Shao, Aimei Song, Fan Zhang

**Affiliations:** 1 Department of Orthodontics, School and Hospital of Stomatology, Cheeloo College of Medicine, Shandong University & Shandong Key Laboratory of Oral Tissue Regeneration & Shandong Engineering Laboratory for Dental Materials and Oral Tissue Regeneration & Shenzhen Research Institute of Shandong University, Jinan, Shenzhen, China; 2 Changzhou Hospital of Traditional Chinese Medicine, Changzhou, China; 3 Department of Orthodontics & Department of Periodontology, School and Hospital of Stomatology, Cheeloo College of Medicine, Shandong University & Shandong Key Laboratory of Oral Tissue Regeneration & Shandong Engineering Laboratory for Dental Materials and Oral Tissue Regeneration, Jinan, China

## Abstract

**Background:**

The patients of bialveolar protrusion always demonstrate thin anterior alveoli which may aggravate subsequent gingival recession and bone loss during retraction. This study aimed to investigate the periodontal changes, including alveolar height, thickness, and area, and the width of keratinized gingiva, in mandibular anterior teeth after augmented corticotomy-assisted orthodontics (ACAO) compared with traditional orthodontics.

**Methods:**

Twenty adult patients with skeletal class I bialveolar protrusion were selected from two groups: ACAO group (augmented corticotomy on the labial side of the anterior mandibular teeth, n = 10) and control group (conventional orthodontics, n = 10). In all patients, four first premolars were extracted and the incisors were retracted under the maximum anchorage. The measurements included the labial alveolar bone area, vertical alveolar bone height, alveolar bone thickness surrounding the mandibular anterior teeth, root length, gingival recession and width of keratinized gingiva after alignment (T0) and 3 months after space closure (T1).

**Results:**

The labial alveolar height, area, and thicknesses all decreased after space closure in the control group but significantly increased in the ACAO group. The decrease in the lingual alveolar height was statistically less in the ACAO group than that in the control group. Besides, the width of keratinized gingiva increased in the ACAO group but decreased in the control group. There was no significant difference in the changes of root length between groups. The dentoalveolar changes between anterior teeth were consistent but with different scales. The lateral incisors gained the most labial bone height and area.

**Conclusion:**

Compared to conventional orthodontics, ACAO provided a more favorable effect of improving periodontal status surrounding the mandibular anterior teeth for Class I maxillary protrusion patients.

## Introduction

Bialveolar protrusion is one of the common dentofacial deformities in Asian populations [1]. Its typical orthodontic treatment is the extraction of four first premolars to retract and/or up-right the maxillary and mandibular incisors to improve the facial profile of patients. Adult patients with bialveolar protrusion usually have high treatment expectations, demanding the most retraction to achieve a straight profile [2]. However, the compromised periodontal status in adult might restrict our access to the best treatment. Firstly, marginal bone loss and gingival recession are the potential periodontal risks when large-scale retraction is performed in adults [3]. In particular, patients with bialveolar protrusion demonstrate thin and elongated upper and lower anterior alveoli [4], and dehiscence and fenestration are commonly seen in their naturally occurring alveolar bone, especially in the lower anterior region [5]. Besides, the tissue response of adults to orthodontic forces including cellular activity and conversion of collagen fibers is less reactive compared to adolescent patients [2], resulting in worse periodontal remodeling. All these may aggravate subsequent gingival recession and bone loss during tooth movement [6, 7].

Therefore, to pursue an efficient tooth movement with improved periodontal remodeling, the augmented corticotomy-assisted orthodontics (ACAO) has been reported to be advantageous [7–12]. It includes selective alveolar corticotomy, bone grafting, and orthodontic forces [13]. Many studies concluded that ACAO facilitated the faster movement of teeth and the increase of alveolar volume [7–12]. However, evidence-based research found that there is still lacking high-quality studies to draw valid and comprehensive conclusions on the periodontal changes caused by ACAO [14–16]. The periodontal conditions are pivotal for long-term stability and patient satisfaction after orthodontics. For instance, the alveolar bone dehiscence may result in gingival recession and reduction of keratinized gingiva, which may increase the incidence of periodontitis [17]. Periodontitis can further activate the nod-like receptor family pyrin domain-containing protein-3 (NLRP3) complex inflammasome in serum and saliva and thus aggravate the condition [18]. The dentoalveolar effect after augmented corticotomy in Class III patients has been well reported to assist the decompensation of the mandibular anterior teeth [7, 8, 12, 19, 20]. However, there were few reports about the dentoalveolar changes of ACAO in the treatment of bialveolar protrusion patients. Therefore, it would be beneficial to dentists to clarify the periodontal effect of ACAO compared to conventional orthodontic treatment in patients with bialveolar protrusion.

To this end, the objective of this study was to evaluate the effect of ACAO on alveolar bone and gingiva in the management of bialveolar protrusion in adult patients. The changes of alveolar bone (the height, area, and thickness) and root resorption in lower anterior teeth were quantitatively measured by the cone-beam CT (CBCT). The soft tissue changes were evaluated by clinical measurement of the gingiva recession and the width of keratinized gingiva. The hypothesis was that the changes of alveolar bone and gingiva in the lower anterior teeth are favorable after ACAO compared with conventional procedures.

## Materials and methods

### Subjects and samples

A retrospective study was designed. All procedures performed in this study involving human participants were approved by the Medical Ethics Committee of the School of Stomatology, Shandong University, Jinan, China (permit NO.20180703) and were in accordance with the Declaration of Helsinki for research involving human subjects. The research was explained to the participants, and a written consent was obtained from each patient.

The subjects included 20 adult patients characterized by Class I bialveolar protrusion who underwent orthodontic treatment from 2016 to 2018 in the Department of Orthodontics, Hospital of Stomatology, Shandong University. The patients were divided into two groups according to the application of augmented corticotomy: ACAO group (with augmented corticotomy, n = 10 [6 women, 4 men]; mean age, 23.9 ± 3.64 years) and control group (without augmented corticotomy, n = 10 [7 women, 3 men]; mean age, 21.4 ± 4.3 years). The primary outcome is the thicknesses gain in the middle part of alveolar bone. Based on a preliminary analysis, a sample size of 10 subjects was calculated to achieve 80% power to detect a difference of 1.1 mm (SD 0.8) between control and treatment groups [19]. All patients received orthodontic treatment from Dr. F. Zhang and periodontal surgery from Dr. A. Song.

Patients inclusion criteria:

1. Adults: age ≥ 18 years old;
2. Class I skeletal profile: SNA (°) = 82.8° ± 4.0°, SNB (°) = 80.1° ± 3.9°, and ANB (°) = 2.7° ± 2.0;
3. Protruded front teeth: U1-NA (mm) > 7.6 mm; L1-NB (mm) > 8.8 mm; U1-L1 (°) < 118°; (4) cases with mild dental crowding (0–4 mm);
4. Cases requiring the extraction of four first premolars to retract and up-right the maxillary and mandibular incisors;
5. Cases requiring maximum anchorage;
6. Healthy subject with no preexisting periodontitis.

Exclusion criteria:

1. Periodontally compromised cases;
2. Presence of preexisting root resorption;
3. History of orthodontic treatment;
4. Cases with moderate to severe crowing (> 4 mm);
5. Patients using bisphosphonates or nonsteroidal anti-inflammatory drugs or on hormone replacement therapy;
6. Patients with endodontically treated anterior teeth.

### Treatment procedures

After the extraction of four first premolars, all patents received MBT™ fixed appliance orthodontic bracket system. During the alignment and arch leveling, sequential arch-wires were engaged involving 0.014-in, 0.016-in, 0.018-in, and 0.019 × 0.025-in nickel-titanium wires, followed by a 0.019 × 0.025-in stainless steel wire. After alignment was completed, CBCT images of each patient were obtained. Thereafter, all patients received implantation of mini-implants (Cibei Co. Ltd., Ningbo, China) on the buccal aspects of the bilateral maxillary and mandibular posterior teeth to provide maximum anchorage.

**Fig. 1:**
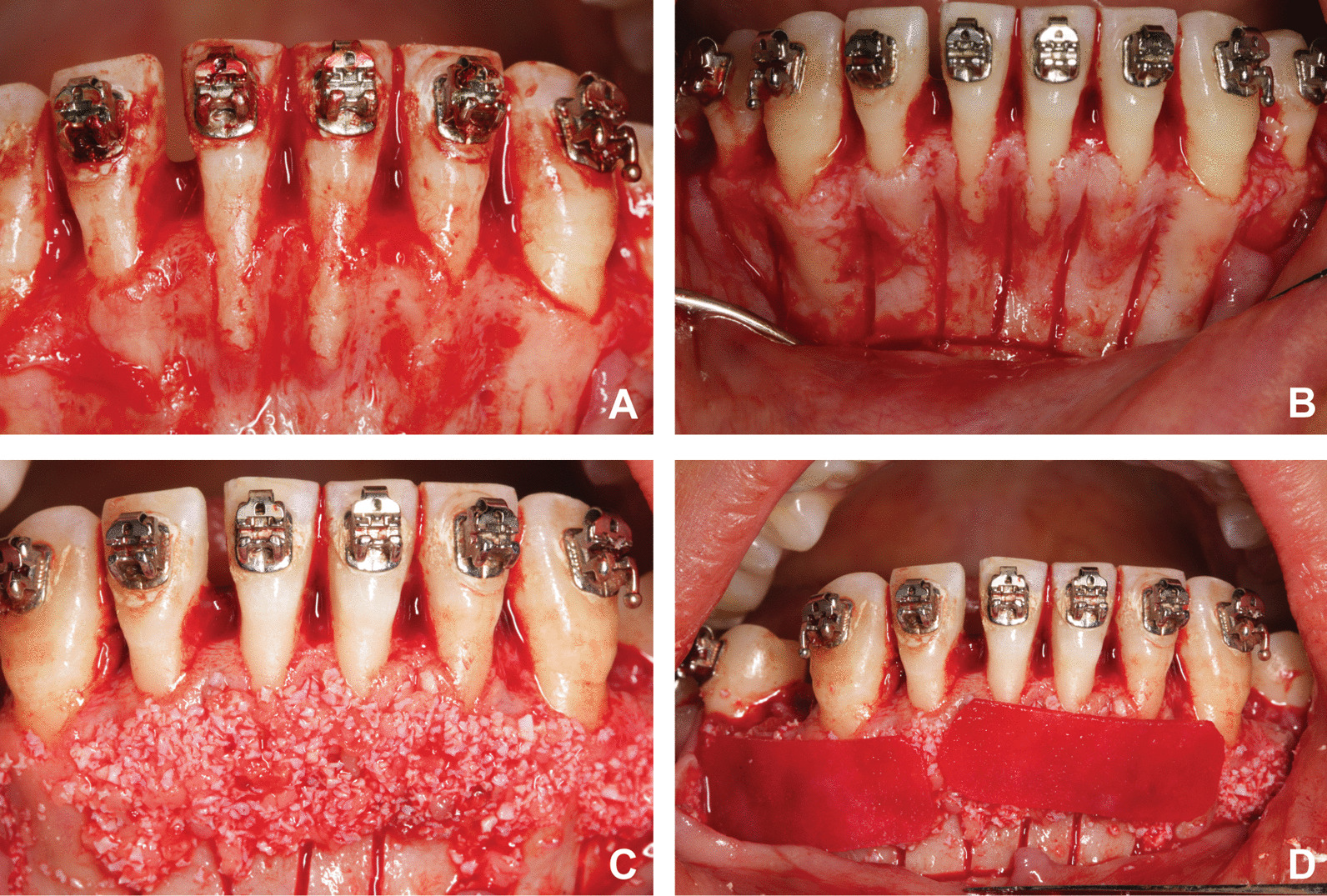
The description of augmented corticotomy surgery. **A** Reflection of a full-thickness flap in the lower anterior region. **B** Perform selective alveolar decortication. **C** Place bovine inorganic bone over the anterior region. **D** Apply the collagen membranes over the bone graft materials

Patients in the ACAO group accepted the augmented corticotomy surgery which was performed by a periodontal specialist after one week of the alignment of all teeth. The surgery procedure started with the reflection of a full-thickness mucoperiosteal flap combined with vertical releasing incisions in the buccal area from the left second premolar till the right second premolar (Fig. 1A). Subsequently, alveolar decortications penetrating the medullary bone were conducted around the lower anterior teeth (Fig. 1B). Bovine inorganic bone granules (Bio-Oss, Geistlich Biomaterials AG, Wolhuser, Switzerland) were then placed over the wounded alveoli (Fig. 1C). Thereafter, collagen membranes (Bio-Guide, Geistlich Biomaterials AG) were trimmed and covered the bone grafting area (Fig. 1D). Finally, the tissue flap was sutured in its original position with non-resorbable suture material. Retraction started immediately after the surgery. In all cases, the retraction was performed with sliding mechanics using e-chains which were changed at 2-week intervals for the ACAO group and 4-week for the control group. The spent period of space closure was recorded in each patient.

### CBCT image acquisition and measurement parameters

Cone-beam computed tomography (CBCT) images (NewTom VG, Aperio Services, Italy) of both groups were obtained at alignment and arch leveling (T0) and 3 months after the closure of the space in the mandible (T1).

**Fig. 2:**
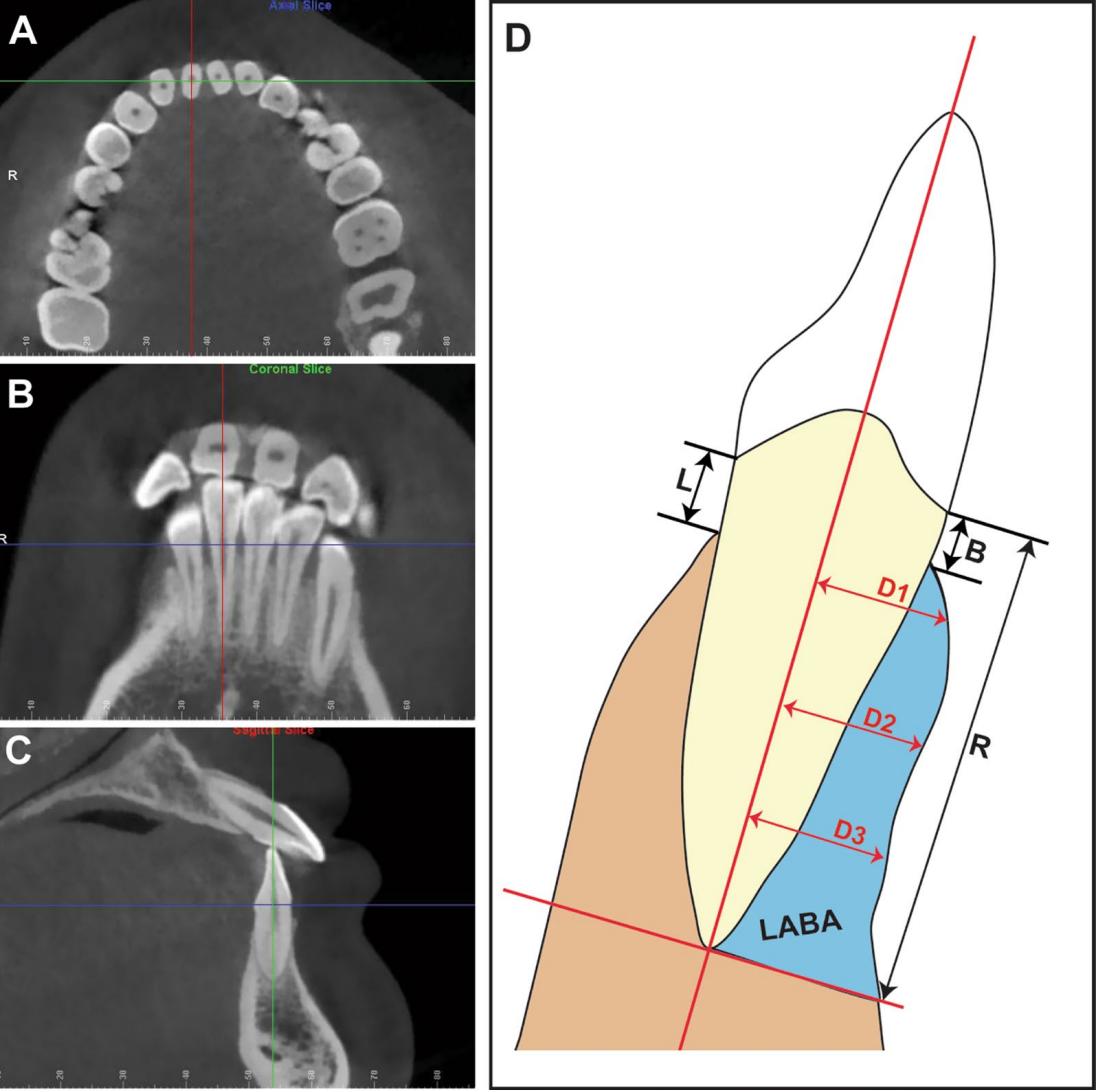
Representative images presenting the protocol of measurement. The correlated planes were determined by 3 intersected guidelines with different colors, which are blue for an axial plane, red for a sagittal plane, and green for a coronal plane. **A** Adjust the location of the axial plane by passing the blue guideline through the CEJ of the selected tooth in both the coronal and sagittal views, then rotate the green guideline until the intersecting line is the shortest. **B** Rotate the red guideline until it passes through the root apex and the midpoint of the incisal margin. **C** Rotate the green guideline until it passes through the root apex and the cusp. **D** Variables measured in CBCT in the sagittal plane. 1. LABA, the blue alveolar bone area of the labial side of the sagittal plane of the 6 mandibular anterior teeth. 2. vertical alveolar bone loss (CEJ-crest, the distance from the cementoenamel junction of the 6 mandibular anterior teeth to the alveolar bone crest measured on the sagittal plane); B, labial bone loss; L, lingual bone loss. 3. R, root length (distance from the cementoenamel junction of the 6 mandibular anterior teeth to the root apex, measured parallel to the long axis of the tooth on the sagittal plane); 4. upper (D1), middle (D2), and lower (D3) alveolar thicknesses (labial alveolar thickness on the sagittal plane as a distance from the root surface of the 6 mandibular anterior teeth to the labial surface of the alveolar bone, measured perpendicular to the long axis of the tooth at 3 mm, 6 mm, 9 mm below the CEJ)

The acquired CBCT data were processed in Dolphin software (Dolphin 11.8, Chatsworth, CA, resolution 0.01 mm), and the bilateral lower incisors and canines of each patient were chosen for the measurements. The CBCT raw data of both groups were randomly mixed before evaluation and the examiner was blinded to the group assignment. Intra-examiner reliability was ensured by reading all images by the same examiner and repeating the same measurement procedure after 2 weeks. The measurement plane was defined as the largest labiolingual section of each tooth. The parameters were the labial alveolar bone area (LABA), vertical alveolar bone level on the labial (B) and lingual (L) sides, root length (R), and upper (D1), middle (D2), and lower (D3) alveolar bone thicknesses on the labial side. The detailed protocol of CBCT measurements was modified based on previous studies [7, 12, 19], which was described in Fig. 2.

### Evaluation of clinical periodontal parameters

The key periodontal parameters to evaluate clinical healing were recorded at both T0 and T1 by one orthodontist:

1. Gingival recession (GR): record the number of teeth with GR in each group. GR is defined as that the gingival margin of the labial surface is under the cementoenamel junction (CEJ).
2. The width of keratinized gingiva (WKG): the distance between the coronal edge of the gingival margin to the mucogingival junction, measured at the mesial, middle and distal points of the labial surface of the tooth.

## Statistical analysis

All data are given as mean ± standard deviation (SD). The observations in each group were normally distributed. The intra-examiner error was tested by a paired Student’s *t-*test, and the intraclass correlation coefficient (ICC) was further calculated. Subsequently, a paired Student’s *t-*test was performed to compare the changes between baseline (T0) and after retraction (T1) in each group, i.e. intragroup difference. Thereafter, the changes between T0 and T1 were calculated separately, shown as d_mean_ and d_SD_, in each group. A Student’s *t*-test for independent samples was performed to compare the changes between the ACAO and the control group. One-way analysis of variance with the Tukey multiple comparison was used to compare the difference between the anterior teeth. All statistical analyses were done on SPSS version 19.0 for Window package (IBM Corp., Armonk, NY). The significance level was set at a 2-tailed *P* value of 0.05 for all tests.

## Results

After the treatment, all protruded anterior teeth of the 20 patients were retracted back to the normal range. The total retraction period is 10.7 ± 1.91 months in ACAO group, which was shorter than that 15.1 ± 3.23 months in control group (*P* < 0.05). In total, 120 lower anterior teeth were measured, including 60 (20 central incisors, 20 lateral incisors, and 20 canines) in each group. The evaluation of the hard and soft tissue before and after the retraction was reported as below.

### Hard tissue evaluation

Regarding the reproducibility of the CBCT measurements, the intra-examiner error was not significant (*P* > 0.05), and ICC was above 0.90. When comparing the baseline level of all parameters between the ACAO and the control groups, no statistical difference was found in any comparison, suggesting an equal baseline in both groups.

#### Root length

After the retraction, slight root resorption of around 1 mm was detected in both groups before and after the treatment (*P* < 0.05), as shown in Table 1. Meanwhile, no statistical difference was detected between the two groups.

**Table 1:** The changes of the root length before and after the retraction

ACAO group			Control group			d_ACAO_ vs d_control_ *p*
T0 Mean ± SD (mm)	T1 Mean ± SD (mm)	T1 versus T0 *P*	T0 Mean ± SD (mm)	T1 Mean ± SD (mm)	T1 versus T0 *P*	
12.69 ± 2.20	11.72 ± 2.29	**0.02**	13.28 ± 1.48	12.25 ± 2.36	**0.005**	0.912

The significance level* P* < 0.05 was labeled bold

d: the difference of the root length between T1 and T0

#### Labial alveolar bone area (LABA)

As shown in Table 2, for the mandibular canines, the LABA slightly increased by 2.37 ± 2.30 mm^2^ in the ACAO group, but no significant change was found in the control group. As for the lateral and central incisors, the LABA increased by 4.80 and 3.70 mm^2^ respectively in the ACAO group (both *P* < 0.01), however, it decreased over 3 mm^2^ in the control group (both *P* < 0.01).

**Table 2:** Alveolar bone changes surrounding mandibular anterior teeth before and after the retraction

	ACAO group				Control group				**d**_**ACAO**_ **vs** **d**_**control**_ P
	T0 Mean ± SD	T1 Mean ± SD	T1 versus T0 *P*	d_ACAO_ Mean ± SD	T0 Mean ± SD	T1 Mean ± SD	T1 versus T0 *P*	**d**_**control**_ Mean ± SD	
*Canines*									
LABA (mm^2^)	10.54 ± 1.51	12.91 ± 1.74	**0.000**	2.37 ± 2.30	9.94 ± 2.04	10.43 ± 1.63	0.407	0.49 ± 2.61	0.021
B (mm)	3.45 ± 0.85	1.34 ± 0.80	**0.000**	− 2.11 ± 1.16	3.43 ± 0.60	4.25 ± 0.63	**0.000**	0.82 ± 0.87	**0.000**
L (mm)	3.04 ± 0.47	3.84 ± 0.66	**0.000**	0.80 ± 0.81	2.45 ± 0.41	4.19 ± 0.73	**0.000**	1.74 ± 0.84	**0.001**
D1 (mm)	4.06 ± 0.96	4.49 ± 0.91	0.154	0.43 ± 1.32	4.38 ± 0.82	4.05 ± 0.67	0.127	− 0.33 ± 1.06	0.052
D2 (mm)	3.49 ± 0.66	4.05 ± 0.83	**0.023**	0.56 ± 1.06	3.73 ± 0.56	3.65 ± 0.62	0.671	− 0.08 ± 0.84	**0.041**
D3 (mm)	3.15 ± 0.29	3.73 ± 0.53	**0.000**	0.58 ± 0.60	3.47 ± 0.65	3.36 ± 0.47	**0.543**	− 0.11 ± 0.80	**0.004**
*Lateral incisors*									
LABA (mm^2^)	9.08 ± 1.33	13.88 ± 1.56	**0.000**	4.80 ± 2.05	9.91 ± 1.99	6.68 ± 1.49	**0.000**	− 3.23 ± 2.49	**0.000**
B (mm)	3.91 ± 0.82	1.34 ± 0.45	**0.000**	− 2.57 ± 0.94	3.66 ± 0.58	4.42 ± 0.68	**0.001**	0.76 ± 0.89	**0.000**
L (mm)	3.31 ± 0.57	4.41 ± 0.54	**0.000**	1.10 ± 0.79	3.61 ± 0.63	5.29 ± 0.64	**0.000**	1.68 ± 0.90	**0.036**
D1 (mm)	3.57 ± 1.16	3.66 ± 1.02	0.796	0.09 ± 1.54	3.68 ± 1.07	3.56 ± 0.97	0.712	− 0.12 ± 1.44	0.660
D2 (mm)	3.10 ± 0.56	3.91 ± 0.60	**0.000**	0.81 ± 0.82	3.56 ± 1.12	3.24 ± 0.66	0.278	− 0.32 ± 1.3	**0.002**
D3 (mm)	2.51 ± 0.53	3.74 ± 0.63	**0.000**	1.23 ± 0.82	3.48 ± 0.51	2.99 ± 0.48	**0.003**	− 0.49 ± 0.70	**0.000**
*Central incisors*									
LABA (mm^2^)	10.76 ± 0.64	14.46 ± 1.01	**0.000**	3.70 ± 1.20	10.67 ± 1.61	6.86 ± 1.52	**0.000**	− 3.81 ± 2.21	**0.000**
B (mm)	2.50 ± 0.54	1.38 ± 0.39	**0.000**	− 1.12 ± 0.67	2.61 ± 0.64	3.35 ± 0.58	**0.001**	0.74 ± 0.86	**0.000**
L (mm)	2.98 ± 0.64	4.63 ± 0.78	**0.000**	1.65 ± 1.01	1.92 ± 0.44	4.34 ± 0.68	**0.000**	2.42 ± 0.81	**0.011**
D1 (mm)	2.95 ± 1.21	3.22 ± 1.26	0.494	0.27 ± 0.75	3.19 ± 0.97	3.06 ± 0.99	0.677	− 0.13 ± 1.39	0.427
D2 (mm)	2.54 ± 0.79	3.13 ± 0.65	**0.014**	0.59 ± 1.02	3.02 ± 0.52	2.62 ± 0.63	0.035	− 0.40 ± 0.82	**0.002**
D3 (mm)	2.15 ± 0.33	2.91 ± 0.44	**0.000**	0.76 ± 0.55	3.04 ± 0.58	2.68 ± 0.40	**0.028**	− 0.36 ± 0.70	**0.000**

The significance level* P* < 0.05 was labeled bold

d: the difference of the variables between T1 and T0

#### Vertical alveolar bone loss

For both the labial (B) and lingual (L) sides of the mandibular canines (Table 2), there was a significant vertical alveolar bone loss for about 0.82 and1.74 mm in the control group. In the ACAO group, the bone height loss was only found in the lingual side for 0.80 ± 0.81 mm, but the loss was significantly less than that in the control group (*P* < 0.01). There was a 2.11 ± 1.16 mm gain in the labial bone crest in the ACAO group, which was statistically significant.

A similar trend of changes in B and L was observed in lateral and central incisors (Table 2). Specifically, in the control group, average bone loss was 0.76 mm labially and 1.68 mm lingually for lateral incisors; 0.74 mm labially, and 2.42 mm lingually for central incisors. For the ACAO group, lingual bone loss was 1.10 mm and 1.65 mm separately for lateral and central incisors, both of which were statistically less compared to the lingual bone loss in the control group. Besides, lateral and central incisors gained labial bone height for about 2.57 mm and 1.12 mm separately.

#### Sagittal alveolar thickness

As for the upper alveolar thickness (D1) (Table 2), the data displayed slight increase in the ACAO group and little decrease in the control group in all three kinds of anterior teeth. However, neither the increase nor the decrease was statistically insignificant. Regarding the middle (D2) and lower (D3) alveolar thickness, it significantly increased in all anterior teeth in the ACAO group (all *P* < 0.05), namely D2 was 0.56 ± 1.06 mm (canines), 0.81 ± 0.82 mm (lateral incisors), and 0.59 ± 1.02 mm (central incisors); D3 was 0.58 ± 0.604 mm (canines), 1.23 ± 0.82 mm (lateral incisors), and 0.76 ± 0.55 mm (central incisors). On the contrary, in the control group, D2 was only shown reduction of 0.4 mm in the central incisors. D3 was significantly decreased in lateral incisors by 0.49 ± 0.70 mm, and in central incisors by 0.36 ± 0.70 mm. Overall, the ACAO group got a sagittal alveolar increase mainly on the middle and lower parts compared to the control group.

#### Comparisons between canines, lateral incisors, and central incisors in the ACAO group

**Fig. 3:**
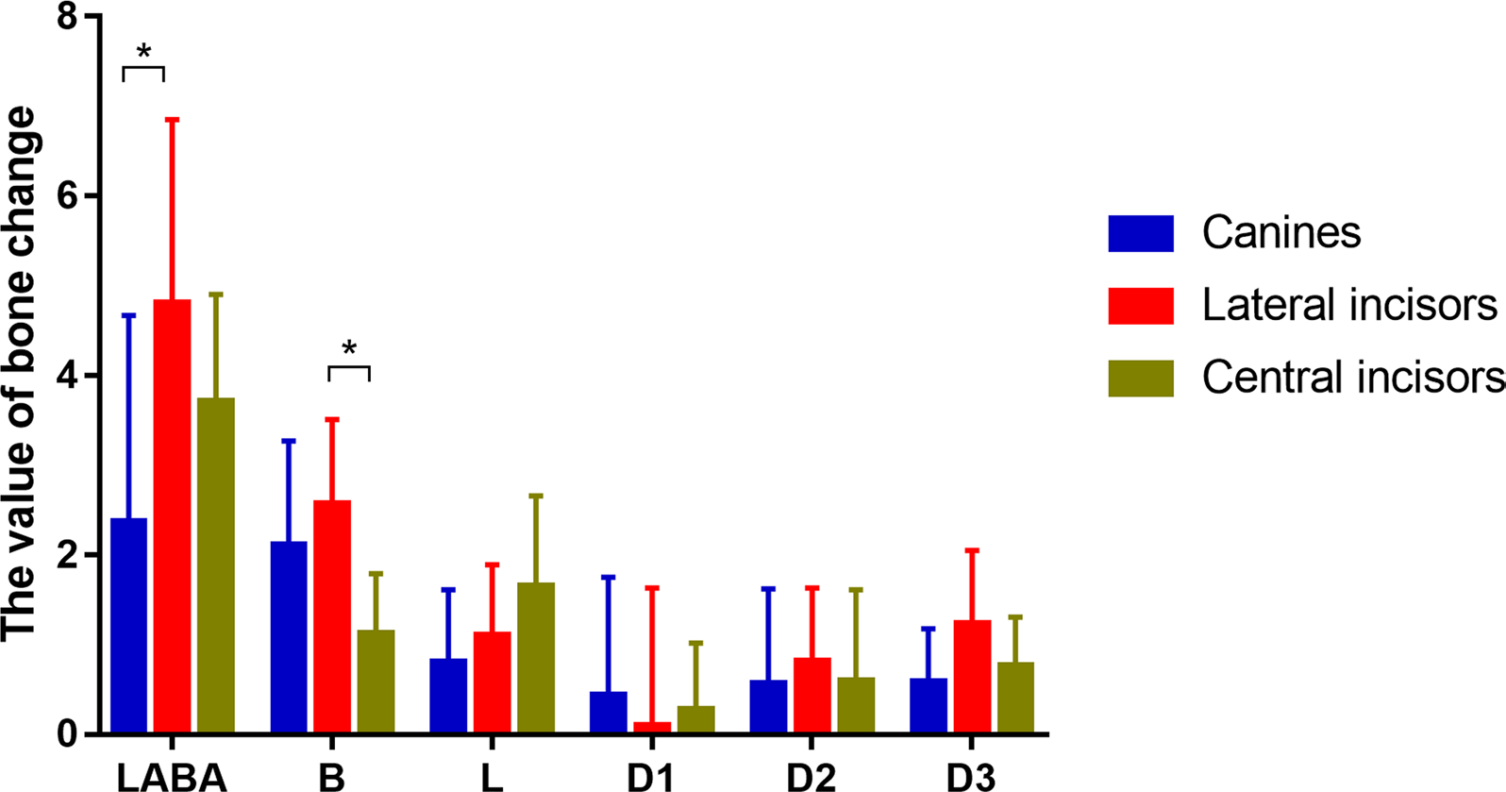
The comparisons on bone change between the three kinds of anterior teeth in the ACAO group. The unit of bone change of LABA is mm^2^. The unit of B, L, D1, D2 and D3 is mm. * indicates *P* < 0.05

As shown in Fig. 3, when comparing the three kinds of anterior teeth after applying augmented corticotomy, the lateral central incisors gained more LABA than the canines. Regarding the vertical bone loss, all had bone height gain labially (B) and bone height loss lingually (L). The labial bone gain was more in the lateral incisors than in the central incisors. The difference of lingual bone loss was not significant among the different teeth. When comparing the sagittal alveolar thicknesses on the labial side, no difference was detected between the three kinds of teeth.

### Clinical evaluation

#### Gingival recession (GR)

In the control group, there were 4/60 teeth showed preexisting GR on average before retraction. Similarly, 5/60 teeth presented GR in the ACAO group. After retraction, 6 newly developed GR was discovered in the control group, and no new GR found in the ACAO group.

#### The width of keratinized gingiva (WKG)

Table 3 displayed the changes of WKG before and after space closure. In both ACAO and control groups, the level of WKG at baseline (T0) was similar (*P* > 0.05, not shown in the table). It was significantly increased at T1 in the ACAO group, with an average of 0.48 ± 1.84 mm. Whereas, decrease of 0.45 ± 1.76 mm of WKG was found in the control group. Further analysis revealed that the changes in WKG were significant between the ACAO and the control groups.

**Table 3:** The changes of the width of keratinized gingiva before and after the treatment

ACAO group			Control group			d_ACAO_ versus d_control_ *P*
T0 Mean ± SD (mm)	T1 Mean ± SD (mm)	T1 versus T0 *P*	T0 Mean ± SD (mm)	T1 Mean ± SD (mm)	T1 versus T0 *P*	
2.42 ± 1.60	2.90 ± 0.91	**0.046**	2.47 ± 1.43	2.02 ± 1.02	0.050	**0.006**

The significance level* P* < 0.05 was labeled bold

d: the difference of the width of keratinized gingiva between T1 and T0

## Discussion

For bialveolar protrusion malocclusion, to reduce the facial convexity, long-distance retraction of the maxillary and mandibular incisors is required. However, large amounts of movement of anterior teeth, especially lower anterior teeth, addresses great risk to develop marginal bone loss and gingival recession. The current study quantitatively evaluated the changes of alveolar bone, root length, and gingival tissue after ACAO treatment compared with traditional orthodontics. Our results showed that the ACAO treatment significantly improved labial alveolar height, thickness, area, and the width of keratinized gingiva compared to traditional orthodontics. Only slight root resorption (≈ 1 mm) was found in both groups.

Several studies have reported alveolar bone loss after orthodontic treatment, mainly on the mandibular anterior teeth under long forward or backward movement [21, 22]. This is consistent with our results: after traditional treatment, both vertical alveolar height and sagittal bone thickness were decreased. However, several clinical studies claimed that vertical bone loss also occurred in the augmented corticotomy group [8, 20]. One possible reason might be the deposition of the implanted bone graft materials in the apical third area, resulting in limited effect on the upper alveolar part. However, in this study, the augmented corticotomy with bovine bone sealed with collagen membranes was proved to increase the labial alveolar height in all lower anterior teeth. This result confirmed the success of the surgery. The histological examination in animals has suggested that application of collagen membranes in augmented corticotomy could promote the planting of a mesenchymal matrix on the bone surface so as to enhance periodontal tissue regeneration [23]. Furthermore, the upper alveolar bone thickness was well preserved and the middle and lower alveolar thickness greatly increased in the ACAO group compared to the control group, which was consistent with the previous research [12].

The large retraction of the anterior teeth might induce severe lingual bone resorption [21]. Our data from the control group revealed more lingual bone height loss than the labial. Therefore, to prevent the potential lingual and labial alveolar loss, meanwhile to solve the preexisting labial dehiscence, we assumed it would be better to perform the augmented corticotomy surgery on both sides [7]. However, due to the traumatic nature of this procedure, most patients did not accept two-sides surgery. Instead, the labial side surgery was adopted. Herein, a dramatic increase in alveolar height, thickness, and area was observed in the labial area of the ACAO group. The lingual alveolar height was decreased as anticipated. Encouragingly, the lingual bone decrease was significantly less in the ACAO group compared to that in the traditional orthodontic group. This indicates that the labial augmented corticotomy surgery exerts some beneficial effects on the lingual periodontal tissue. We speculated this phenomenon resulting from the rapid tooth movement to reduce the retraction phase induced by ACAO [9], which eventually promoted the improvement in the general periodontal stability.

Slight root resorption was detected in both groups but with no statistical significance between the groups. Despite several have reported root resorption after using bone graft materials in ACAO [24, 25], most studies did not find a correlation between ACAO and root resorption [7, 12, 15].

Nimigean et al. reported that dehiscence occurred more in the mandibular canines after examining 138 dry skulls of adults [26]. Since the position and morphology of incisors and canines in the dentition are quite different, we speculated the effect of augmented corticotomy might be different. Our results showed that a favorable response of augmented corticotomy was observed in all anterior teeth. The total dentoalveolar changes in the three kinds of anterior teeth were comparable. No significant difference was detected. This result was in line with the data from a previous study, where the augmented corticotomy to the mandibular anterior teeth resulted in similar dentoalveolar change between the incisors and the canines [12].

Regarding the soft tissue in periodontal healing, the width of keratinized gingiva was decreased in the control group while increased in the ACAO group. Meanwhile, some newly developed gingival recession was detected in the control group but not in the ACAO group. It is evident that the healing pattern of soft tissue is consistent with the changes in hard tissue. These results also corroborate Wilcko’s observation [10]. They found a width increase of 0.78 mm on the keratinized gingiva at 1.5 years after the augmented corticotomy assisted orthodontic treatment. Therefore, the ACAO technique is favorable in the remodeling of both hard and soft tissue. Additionally, it is worth mentioning that gingival phenotype might be another factor to consider when evaluating the periodontal healing after orthodontic treatment. According to research from Gaetano Isola et al*.*, they found females patients of malocclusion presented higher prevalence of thick gingival biotypes [27]. Therefore, in future studies, it is interesting to investigate the association of gingival biotype and periodontal healing following corticotomy.

This clinical study also has several limitations. First, periodontal remodeling normally continues until 1–2 years after debonding, and thus long-term stability is paramount for successful orthodontic treatment. The effect on augmented bone sites should track for a longer period in future studies. Second, retrospective studies are assumed to have more bias than prospective studies. Even though the registry was strictly executed in the current study, selection bias is inevitable, especially with such a small sample size. Therefore, a randomized control trial is recommended to provide a higher level of evidence. Third, corticotomy is to create an intentional injury that initiated an inflammatory response subsequently with a regenerative process [28]. It is orchestrated and coordinated by different cell types and signaling factors. For example, three weeks after initiation of tooth movement assisted by corticotomy technique, tumor necrosis factor alpha levels and pre-osteoclast and/or osteoclast counts elevated 2–4 times greater [29]. However, prolonged inflammation e.g. increased NLRP3 inflammasome, might hinder the periodontal healing after augmented corticotomy. Thus, to understand the molecular process behind augmented corticotomy-assisted orthodontics, biological molecules that participated in inflammatory and regenerative process of periodontal tissues should be further investigated and discussed.

## Conclusion

Within the conditions of the study, for Class I bialveolar protrusion patients, labial augmented corticotomy in the lower anterior region provides a favorable effect of improving periodontal structure, including the height, thickness, and area of labial alveolar bone and keratinized gingiva surrounding the mandibular anterior teeth, compared with the traditional orthodontics. Besides, the favorable effect of augmented corticotomy was similar between the incisors and canines. Further studies are suggested to investigate if there is a relationship between periodontal biotype, inflammation and corticotomy.

## Data Availability

The datasets used and/or analysed during the current study are available from the corresponding author on reasonable request.
